# Professional and academic pre-qualifications, career preferences and aspirations in working as a rural doctor

**DOI:** 10.3389/fmed.2025.1566303

**Published:** 2025-07-09

**Authors:** Carla Schröpel, Teresa Festl-Wietek, Anne Herrmann-Werner, Tim Wittenberg, Marina Pumptow, Sabine C. Herpertz, Andrea Heinzmann, Katrin Schüttpelz-Brauns, Tobias Maria Boeckers, Stephan Zipfel, Rebecca Erschens

**Affiliations:** ^1^Internal Medicine, Department of Psychosomatic Medicine and Psychotherapy, University Medical Hospital Tuebingen, Tuebingen, Germany; ^2^TIME -Tübingen Institute for Medical Education, Medical Faculty, University of Tuebingen, Tuebingen, Germany; ^3^Department of General Psychiatry, Centre for Psychosocial Medicine, University of Heidelberg, Heidelberg, Germany; ^4^University Hospital Tuebingen - Institute for Clinical Epidemiology and Applied Biometry, Tuebingen, Germany; ^5^Office of the Dean of Studies, Medical Faculty, Albert-Ludwigs-University Freiburg, Freiburg, Germany; ^6^Medical Education Research Department, Division for Study and Teaching Development, Medical Faculty Mannheim at Heidelberg University, Mannheim, Germany; ^7^Office of the Dean of Studies, Medical Faculty, Ulm University, Ulm, Germany; ^8^Deanery of Students’ Affairs, University's Faculty of Medicine, Tuebingen, Germany

**Keywords:** specialty choice, rural doctor, professional and academic pre-qualifications, vocational interests, RIASEC

## Abstract

**Introduction:**

Internationally, countries are struggling to provide health care in rural areas. In Germany, where the medical school’s admissions system rewards prior experience, there are a significant number of students with professional experience (e.g., paramedics, nurses). To date, there has been little research on this pre-experienced subgroup. In a rather exploratory approach, preferences for specialty training were compared between students with and without pre-qualifications. The primary aim of the study was to analyze how pre-qualifications and career types according to Holland’s RIASEC (acronym for realistic, investigative, artistic, social, enterprising, conventional) model contribute to interest in working as a rural doctor.

**Methods:**

Overall, 2,370 medical students at different stages of their studies (i.e., 3rd, 6th, 10th semester, and final year) completed the questionnaire. Students indicated interest in working as a rural doctor on a 9-point scale, and expressed interest in up to three specialist training programs from a list of 16. In addition, students answered questions about professional and academic pre-qualifications (i.e., vocational training in the medical field, academic degree, voluntary service) and completed a 6-item questionnaire on vocational interests according to the RIASEC model. The study was a multicenter cross-sectional study conducted at all five medical schools in the federal state of Baden-Württemberg, Germany.

**Results:**

Results show differences in career aspirations according to different pre-qualifications, especially for the pre-qualifications vocational training and voluntary service. The strongest association was found between having completed vocational training and interest in Anesthesiology, OR = 3.92 [3.22, 4.76]. A linear mixed model revealed that higher interest in practical-technical (realistic RIASEC type) or social activities (social RIASEC type), and lower interest in intellectual-research activities (investigative RIASEC type) predicted interest in rural practice, whereas pre-qualifications did not contribute significantly to the model.

**Discussion:**

The findings contribute to a better understanding of the career preferences of medical students with pre-qualifications. Previous experience may lead to the formation of a professional identity and community of practice (CoP) before medical school, which may also influence career preferences. To promote interest in rural medicine, medical schools could encourage interest in social and practical-technical activities within the curriculum and strengthen the profile of general practice.

## 1 Introduction

Internationally, countries are facing a shortage of doctors in rural areas (1–4). Geographical isolation, fewer educational and career opportunities for their families, demanding working conditions or lower prestige can deter doctors from working in underserved rural areas (3, 5). To ensure rural medical care, factors that increase interest in rural practice need to be identified and promoted. In Germany, since the winter semester 2019/20 (start date depends on the federal state), some federal states have introduced a ‘rural doctor quota’ to ensure long-term care in rural areas. Each year, a number of study places are allocated to students who commit to working in an underserved area after graduating from medical school (6). While there has been little research into the effectiveness of this quota, several international studies have identified exposure to rural practice during medical training, e.g., through internships or rural placement programs (7–13) as a key factor, alongside rural background (6, 14–16) or schooling (17–19).

In addition to the practical experience gained during their studies, there may be a significant number of medical students who have gained practical experience prior to medical school, for example by working as a scribe or through vocational training in the medical field (20–22). In Germany, the chances of being accepted to medical school can be increased by having such practical experience (22, 23). However, there is little research on how professional or academic pre-qualifications relate to medical students’ career aspirations, particularly in relation to rural practice (22).

According to Flum and Blustein (24), a key element in making an informed career-choice is the process of vocational exploration, defined as the appraisal of internal attributes (e.g., interests, abilities) and the exploration of external options (e.g., from relevant vocational contexts). This lifelong process overlaps with Erikson’s phases of identity formation (25). Medical students with previous practical or academic experience may be more advanced in both identity formation and career exploration than those without. They may also develop career preferences based on past learning experiences. This is in line with Social Learning Theory (26), which suggests that career decisions result from multiple learning experiences shaped by a complex set of environmental factors and the cognitive-emotional responses to them.

Specialty choice is the product of complex interactions (27–29). When considering theories and models of the development of preferences for specialty training and decisions in general, the influence of past experience is usually taken into account in some way. According to a theory by Bland (30), when choosing a specialty, it is important that there is a fit between the perceived characteristics of a specialty and the student’s career needs. The student’s value system plays a role, as do the expectations of others, experiences before medical school, the student’s own personality and background, and the culture and characteristics of the medical school. Other models also focus on the optimal fit between specialization and personal preferences. Mitchell (31) defines different domains in the decision-making process (personal characteristics, the cognitive lens, the medical school environment and the choice domain). The personal characteristics domain emphasizes the indirect influence of previous experiences (e.g., family background, previous practical experience) as socialization experiences on the choice of specialty, which may play an important role alongside other variables such as attitudes and values, personality and current life circumstances.

There are indications that individuals with completed vocational training in the medical field are more likely to be interested in general practice (6, 32) or anesthesiology (32). Vocational training in the medical field, e.g., for nurses or paramedics (in Germany these professions do not require a university degree), may share some aspects of anesthesia and general medicine, including intensive patient contact and emergency situations. But is there any evidence of a relationship between interest in rural medicine and previous practical experience? There is some indication that interest in or plans for general practice are associated with greater interest in working in rural areas (33, 34). Studies also show, that medical students with pre-qualifications, such as previous practical experience (35) or voluntary work (17), may be more interested in rural practice than those without. However, there is a paucity of research on the relationship between pre-qualifications and medical students’ career aspirations, particularly in relation to a career in rural practice (22).

**Figure 1:**
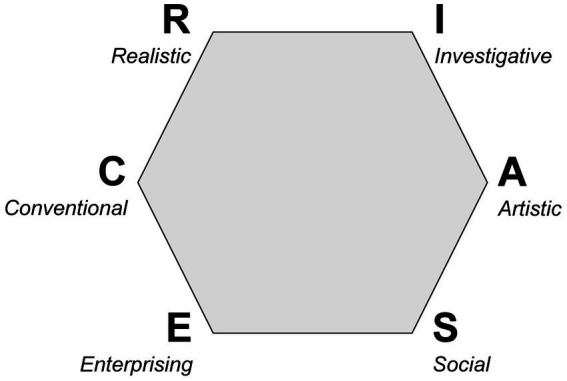
Hexagon of six career types according to Holland’s RIASEC model, adapted from Holland (77).

In order to better understand the career decision-making processes of medical students, it may be beneficial to also consider models of career decision-making in general. Individuals seek professional environments that match their interests (36). In this context, vocational interest tests are a popular career guidance tool (37). A very influential and widely used theory in this context is Holland’s model of vocational interests (38, 39). Individuals and environments can be described by six broad career types, arranged in a hexagonal structure: Realistic, Investigative, Artistic, Social, Enterprising, Conventional - RISAEC (36), see Figure 1. The RIASEC model can be used as an additional source of information in career counseling (40) but it can also provide important information in the decision-making process regarding further specialization within a profession. For example, there are a few studies that examine the RIASEC taxonomy in relation to different medical specialties (41–44). The RIASEC profile results from a letter code, the order of which is determined by the ranking of the values on the six scales. There is evidence that the Holland code for general practice/family medicine may include the social (S; e.g. helping, teaching) (41, 42, 45) and investigative (I; e.g. interest in research, intellectual tasks) components (41, 45). Other studies also suggest an association between a social orientation and career aspirations in general practice (46), including rural practice (17, 18). Although there may be similarities between the work environment of a general practitioner and that of a rural doctor, there is a need for research that explicitly examines the relationship between vocational interests and aspirations to work as a rural doctor.

Despite the increasing importance of rural health care in Germany and the associated support programs such as the rural doctor quota (6), there has been little research on the career aspirations of medical students with professional and academic pre-qualifications. The main focus of our study was to investigate how professional and academic pre-qualifications and vocational interests according to Holland’s RIASEC model relate to interest in working as a rural doctor. Prior to this, a more exploratory approach was used to investigate interest in different specialties among medical students with and without professional and academic pre-qualifications. The present study aims to investigate the following research questions (RQ):

*RQ1*: Are there differences in the preference for different specialty training programs between individuals with and without professional and academic pre-qualifications (i.e. completed vocational training in the medical filed, voluntary service or academic degree)?

*RQ2*: Do academic and professional pre-qualifications relate to interest in working as a rural doctor?

*RQ3*: How do different vocational interests relate to interest in working as a rural doctor?

## 2 Method

### 2.1 Design, sample and procedure

**Figure 2:**
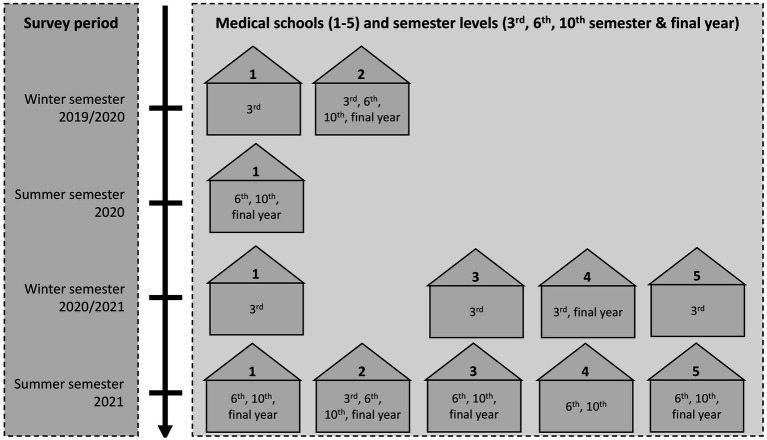
Overview of data collection among medical students at different semester levels across medical schools and survey periods.

This study is a multicenter cross-sectional study conducted at all medical faculties in Baden-Württemberg, a region in south-western of Germany. Medical students at different stages of their studies (i.e., 3rd, 6th, 10th semester, and final year) were recruited via email or during lectures. The questionnaire took about 20 min to complete and was conducted between October 2019 and September 2021. Figure 2 illustrates the data collection process over the four different survey periods in the five medical schools.

### 2.2 Measures

This study is part of the collaborative research network ‘Pre-qualifications for Human Medicine’, examining professional and academic pre-qualifications in the context of medical studies based on clearly defined overarching research questions. Two subprojects analyzing pre-qualifications in relationship to academic success (47) and burnout experience (48) have already been published. The present study is a distinct analysis that examines specialty preferences and interest in rural practice. For this reason, the description of the sample and study procedure in this study may be similar to those in the other subprojects of the aforementioned research network. In the following, we refer to the other publications where necessary.

#### 2.2.1 Sociodemographics and control variables

Medical students provided information about their age, gender and the number of inhabitants in their hometown (rural origin was defined as <5,000 inhabitants). They also indicated whether they had planned or completed an internship in general practice.

#### 2.2.2 Professional and academic pre-qualifications

All medical students responded to questions about their pre-qualifications, namely vocational training in the medical field, an academic degree or voluntary service completed before medical school. We asked in which area the pre-qualification had been acquired and whether the pre-qualification had been completed or not. For a detailed overview of all items and corresponding response formats, please refer to a publication by Schröpel et al. (47). The following three dummy variables (0 = no, 1 = yes) were defined on the basis of the responses of the medical students, and are collectively referred to as ‘professional and academic pre-qualifications’:

1. Vocational training in the medical field, completed with a grade.
2. Academic degree (bachelor/master), completed prior to medical school.
3. Voluntary service (duration of ≥11 months).

#### 2.2.3 Vocational interests

To assess vocational interests according to Holland’s model (36), we utilized a brief scale developed by Bergmann (49). Participants rated their interest in six vocational activities (analogous to the RIASEC taxonomy) on a scale of 1–9, higher scores indicating greater interest. Examples were given for each of the vocational activities (see Table 1).

**Table 1:** Items of Bergmann’s vocational interests scale for measuring interest in six vocational activities according to Holland’s RIASEC model.

Vocational activities	Examples
Practical-technical (R)	e.g., working with machines or technical equipment, working with wood, metal or other materials, repairing equipment or machines.
Intellectual-research (I)	e.g., carrying out experiments, reading scientific literature, solving abstract problems, developing new ideas, making accurate observations, analyzing.
Artistic-linguistic (A)	e.g., drawing, painting, making music, translating, engaging with art and literature, performing in a theater.
Social (S)	e.g., looking after others, teaching, advising, supporting, working with others, caring for others.
Entrepreneurial (E)	e.g., organizing, running a business, supervising others, influencing, leading, promoting something, selling.
Orderly-administrative (C)	e.g., documentation, compiling statistics, conducting correspondence, designing and applying laws and regulations, managing accounts.

The naming of Holland’s RIASEC components (R, realistic; I, investigative; A, artistic; S, social; E, enterprising; C, conventional) slightly differs in Bergmann’s scale.

#### 2.2.4 Preference for specialty training and working as a rural doctor

Medical students could indicate their interest in 15 specialty training programs (plus an ‘other’ option), corresponding to common specialty training programs in Germany. Following the approach of Heinz and Jacob (50), medical students could select a maximum of three programs. To assess interest in working as a rural doctor, medical students indicated their interest on a scale from 1 (not interested at all) to 9 (very interested).

### 2.3 Statistical analysis

For all analyses we used SPSS (28.0.0.0) with a level of significance set at *α* = 0.05. To analyze the associations between different medical specializations with professional and academic pre-qualifications (RQ1), we calculated odds ratio (OR) and corresponding 95% confidence intervals (CI). If the 95% CI for the OR did not include the number 1, we considered the OR to be statistically significant. An OR = 1 would indicate no association, an OR < 1 indicates greater odds in the first group and OR > 1 greater odds in the second group. OR can be an indication of the strength of an association with an OR of about 1.5 indicating a small effect, OR of about 3.0 or more a medium effect, and OR of about 5.0 a large effect (51). To interpret the effect size for OR<1 and compare to reference values, the OR must be inverted (1/OR) (51).

**Figure 3:**
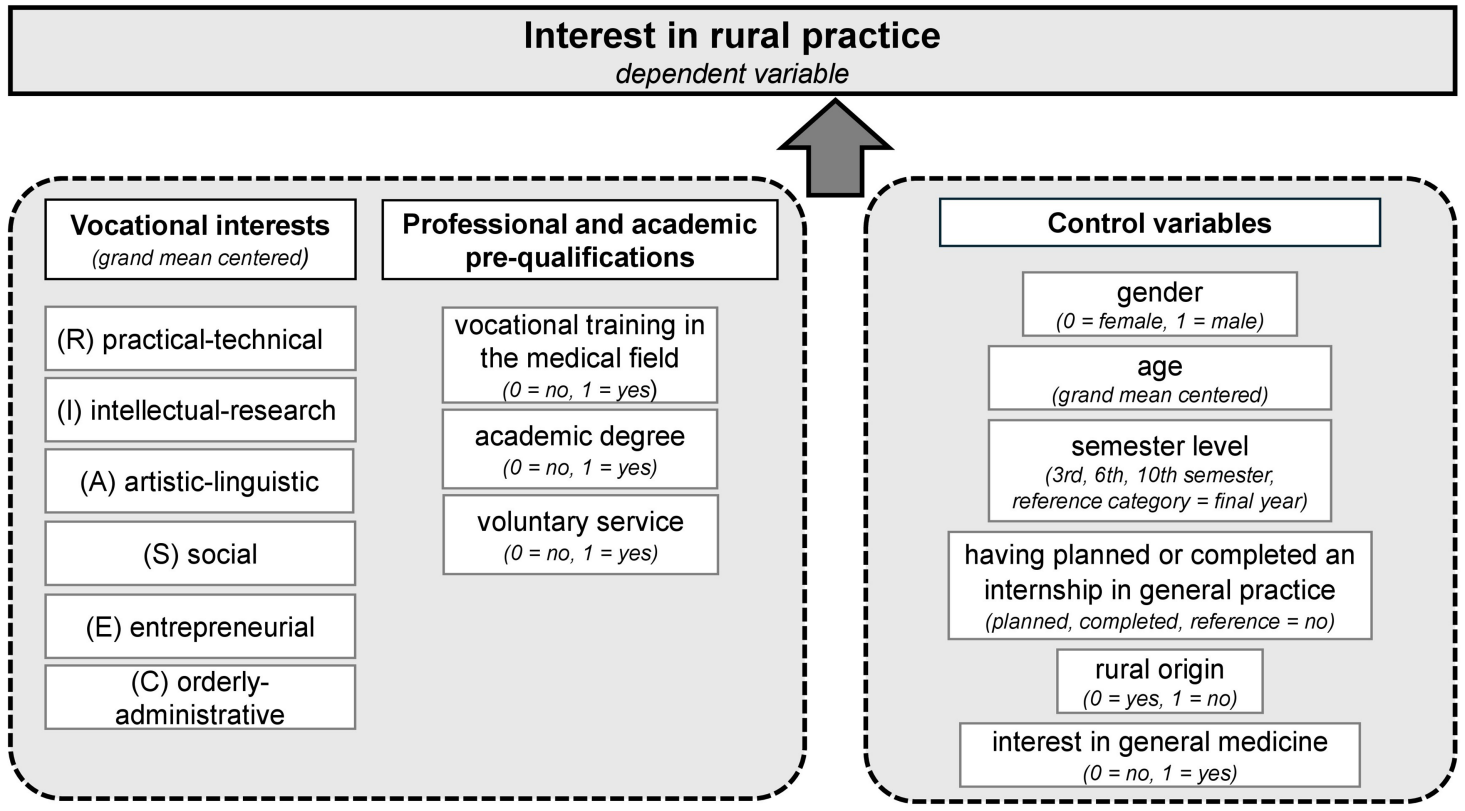
Overview of all predictors included in the regression analysis with interest in rural practice as the dependent variable.

We conducted a linear mixed model, to analyze how professional and academic pre-qualifications (RQ2) and vocational interests (RQ3) relate to interest in working as a rural doctor while accounting for the nested structure of our data. Figure 3 provides an overview of all predictors (including control variables) and their coding, as well as the reference categories of the dummy variables. All continuous predictors were grand mean centered. Estimated marginal means (EMMs) are provided to illustrate the effects. They represent mean values estimated from the regression model, while controlling for the other variables in the model. Before running the analysis, we first calculated intercept-only models and tested whether models with random intercept (random intercept for medical school, survey period or the combination of both) show better fit than the model with fixed intercept. As part of the model selection, we calculated the intraclass correlation coefficient (ICC) and also performed a likelihood ratio test (LRT) to compare the models. The Akaike Information Criterion (AIC) (52) and the Bayesian Information Criterion (BIC) (53), were used as additional parameters for the model selection. Smaller AIC/BIC values indicate a better model fit and are preferred when comparing models. Our study is based on a convenience sample, i.e., we wanted to include as many cases as possible, so no power analysis was calculated prior to the study. However, we conducted a *post hoc* sensitivity analysis using G*Power (54).

## 3 Results

### 3.1 Sample and response rate

Overall *N* = 2,370 medical students took part in our study (women: 64.7%, *n* = 1,521; men: 35.1%, *n* = 826, other: 0.2%, *n* = 4). In total, 635 (26.8%) 3rd-semester students, 729 (30.8%) 6th-semester students, 485 (20.5%) 10th-semester students and 521 (22.0%) students in their final year participated in the study. In total 655 (27.6%) medical students reported having completed vocational training in the medical field (most of them as a paramedic or nurse), 546 (23.1%) students reported having completed a voluntary service (the majority in the health sector) and 145 (6.1%) students reported to have an academic degree (most in a STEM subject or in the medical field). Some medical students reported to have completed two pre-qualifications (vocational training & academic degree, *n* = 8 students; vocational training & voluntary service, *n* = 254 students; academic degree & voluntary service *n* = 10 students) and *n* = 7 students completed all three pre-qualifications. For a detailed description of the sample and medical students’ pre-qualifications see (47).

Overall, 634 (26.8%) medical students reported growing up in a rural area (vs. *n* = 1,732, 73.2% in a larger town or city). A total of 905 (38.7%) students planned to undertake an internship in general medicine, while 1,011 (43.2%) had already completed one and 422 (18.0%) students indicated that they did not plan to do one. Medical students displayed a particular interest in social activities (*M* = 7.30, SD = 1.67), followed by intellectual-research (*M* = 6.16, SD = 2.01) and entrepreneurial activities (*M* = 5.93, SD = 2.10), see Table 2 for a detailed overview of all mean values.

**Table 2:** Interest in the six different occupational activities of Bergmann’s vocational interests scale, sorted by mean.

Vocational activities	*M* (SD)	Mdn
Social (S)	7.30 (1.67)	8
intellectual-research (I)	6.16 (2.01)	6
Entrepreneurial (E)	5.93 (2.10)	6
Artistic-linguistic (A)	5.56 (2.34)	6
Practical-technical (R)	5.23 (2.27)	6
Orderly-administrative (C)	4.83 (2.20)	5

The response format of each scale ranged from 1 to 9, with higher levels indicating greater interest in the corresponding vocational activity.

### 3.2 Preferences for specialty training

We excluded 21 cases for not specifying a specialty or for specifying more than the three allowed. Of all medical students included in the analysis (*N* = 2,349), the majority indicated an interest in Internal Medicine, followed by General Medicine, Pediatrics, Anesthesiology and Surgery, see Table 3. Descriptively, it was found that interest in different specialty training programs varied slightly between semesters. For some specialties the differences were greater, e.g., descriptively, third semester students showed a higher interest in surgery than higher semesters (see Supplementary Figure S1). Medical students could indicate up to three specializations. On average they indicated *M* = 2.63 (SD = 0.64) preferences. Overall, *n* = 445 distinct specialization combinations were identified (see Supplementary Table S1). The most frequent combination was ‘General Medicine–Anesthesiology–Internal Medicine’ (*n* = 84, 3.6%).

**Table 3:** Interest of medical students in common German specialty training programs, sorted by relative frequency.

Specialty training program	Interest in specialty training program *n* (%)	
	Yes	No
Internal medicine	1,037 (44.1)	1,312 (55.9)
General medicine	792 (33.7)	1,557 (66.3)
Pediatrics	645 (27.5)	1704 (72.5)
Anesthesiology	635 (27.0)	1714 (73.0)
Surgery	602 (25.6)	1747 (74.4)
Gynecology and obstetrics	499 (21.2)	1850 (78.8)
Neurology	351 (14.9)	1998 (85.1)
Orthopedics	278 (11.8)	2071 (88.2)
Psychiatry and psychotherapy	227 (9.7)	2,122 (90.3)
Radiology	224 (9.5)	2,125 (90.5)
Psychosomatic medicine and psychotherapy	154 (6.6)	2,195 (93.4)
Dermatology	124 (5.3)	2,225 (94.7)
Urology	119 (5.1)	2,230 (94.9)
Otorhinolaryngology	117 (5.0)	2,232 (95.0)
Ophthalmology	102 (4.3)	2,247 (95.7)
Other	277 (11.8)	2072 (88.2)

Medical students could indicate up to three preferences, *N* = 2,349.

**Figure 4:**
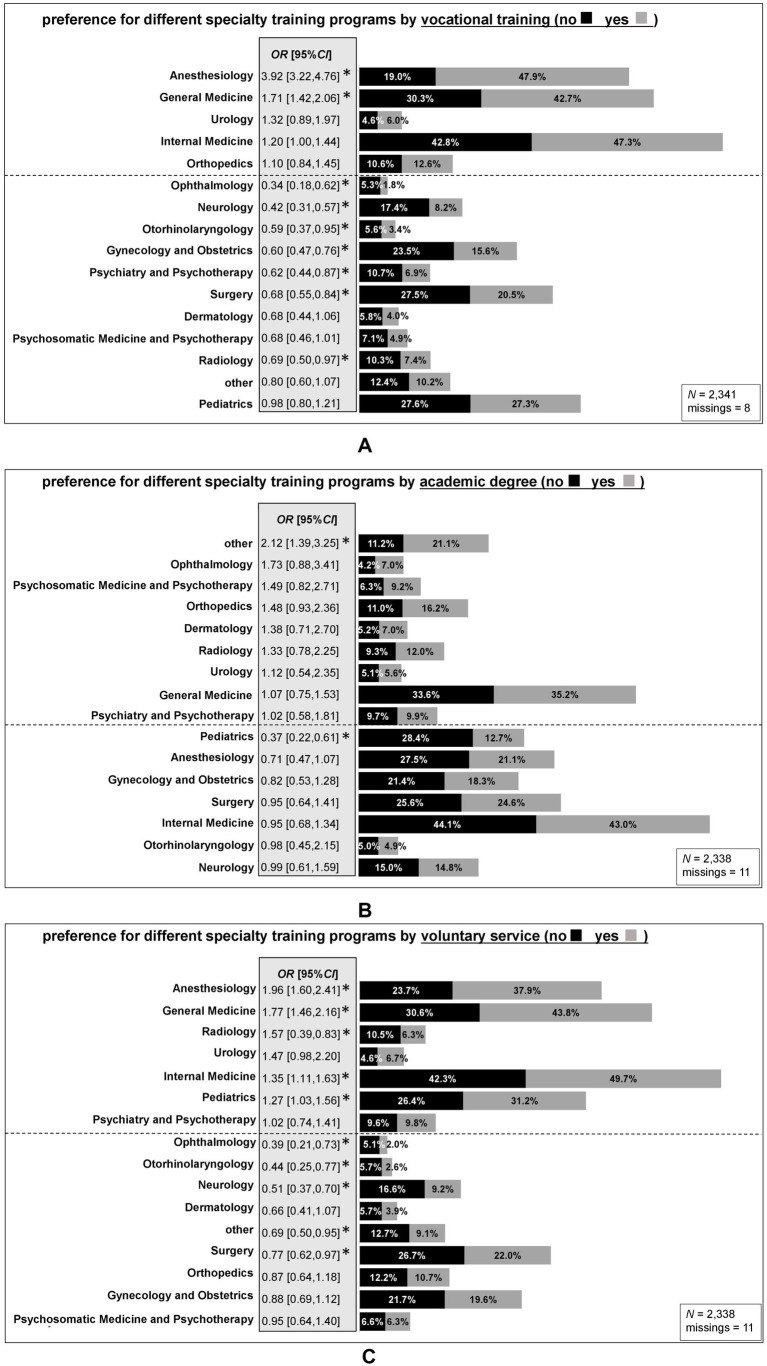
Specialist training preferences of medical students with completed vocational training in the medical field **(A)**, prior academic degree **(B)** or voluntary service **(C)**. The figure shows the percentage of interest in different specialties separately for medical students with and without the corresponding pre-qualification. All associations are sorted by the strength of the association (Odds Ratio, OR). Above the dashed line are OR>1 (higher interest in each specialty for pre-qualified medical students compared to those without pre-qualification), and below the line are OR<1 (lower interest in each specialty for those with the corresponding pre-qualification compared to those without). Statistically significant OR are highlighted with an asterisk (*). Medical students could indicate up to three preferences. OR can indicate the strength of an association (51). As there are only reference values for OR>1 (see statistical analysis, method section), in the following the inverse value for all significant OR<1 is reported. Panel **A**: Ophthalmology (1/OR = 2.94), Neurology (1/OR = 2.38), Otorhinolaryngology (1/OR = 1.69), Gynecology and Obstetrics (1/OR = 1.67), Psychiatry and Psychotherapy (1/OR = 1.61), Surgery (1/OR = 1.47), Radiology (1/OR = 1.45). Panel **B**: Pediatrics (1/OR = 2.70). Panel **C**: Ophthalmology (1/OR = 2.56), Otorhinolaryngology (1/OR = 2.27), Neurology (1/OR = 1.96), other specialty (1/OR = 1.45), Surgery (1/OR = 1.30).

To test for differences in in the preference for different specialty training programs between individuals with and without professional and academic pre-qualifications (RQ 1), we calculated odds ratio (OR) and tested for significance. Figure 4 shows the training preferences for medical students with completed vocational training (panel A), prior academic degree (panel B) and voluntary service (panel C). Specialty training programs above the dashed line and marked with an asterisk were significantly more interesting to medical students with the corresponding pre-qualification than to those without. On the other hand, medical students with the corresponding pre-qualification were less interested in training programs below the dashed line. All OR are sorted according to the strength of the association, and significant OR are marked with an asterisk. In the following, all significant associations are described separately for each of the three pre-qualifications.

The odds of being interested in Anesthesiology (OR = 3.92, medium-large effect) and General Medicine (OR = 1.71, small effect) were higher for medical students with prior vocational training than for those without. In contrast, the odds of being interested in Ophthalmology (OR = 0.34, medium effect), Neurology (OR = 0.42, small-medium effect), Otorhinolaryngology (OR = 0.59, small effect), Gynecology and Obstetrics (OR = 0.60, small effect), Psychiatry and Psychotherapy (OR = 0.62, small effect), Surgery (OR = 0.68, small effect), and Radiology (OR = 0.69, small effect) were lower for students with than without prior vocational training (Figure 4A).

When comparing students with a previous academic degree with those without, two significant differences in specialization preferences were found see (Figure 4B). The odds of being interested in ‘other’ specialty programs (OR = 2.12, small-medium effect), were higher for medical students with previous academic degree than for those without. However, the odds of being interested in Pediatrics (OR = 0.37, small-medium effect) were lower for students with previous degree than for those without.

The odds of being interested in Anesthesiology (OR = 1.96, small-medium effect), General Medicine (OR = 1.77, small effect), Radiology (OR = 1.57, small effect), Internal Medicine (OR = 1.35, small effect), and Pediatrics (OR = 1.27) were higher for students with prior voluntary service than for those without, while the odds of being interested in Ophthalmology (OR = 0.39, small-medium effect), Otorhinolaryngology (OR = 0.44, small-medium effect), Neurology (OR = 0.51, small-medium effect), ‘other’ specialty (OR = 0.69, small effect), and Surgery (OR = 0.77, small effect) were lower for students with prior voluntary service than for those without (Figure 4C).

### 3.3 Interest in working as a rural doctor

We calculated three multilevel null models (maximum likelihood estimation, ML) to test if the intercept of our dependent variable (level 1) was independent of the level 2 grouping variables ‘survey period’ and ‘medical school’ or their combination. All calculated ICC of the null models were close to zero (see Table 4), indicating only a small amount of variance in the dependent variable could be explained due to the variance between the subgroups of the respective level-2 variable (e.g., 4% survey period*medical school). However, the results of the Likelihood Ratio Test indicated that all three random intercept models had a significantly better fit than the intercept-only model with fixed intercept (*p* < 0.001). For the final selection of the model, we compared the information criterions AIC and BIC of the respective models. The intercept-only model with random intercept for the combination of survey period and medical school was found to be the model with the best fit. In the next step we added all predictors to the model and ran the analysis. The final model was estimated using restricted maximum likelihood estimation (REML).

**Table 4:** Model selection, comparison of intercept-only model with fixed intercept and three models with random intercept for survey period, medical school or survey period* medical school.

Model	ICC	−2 LL	LRT	AIC	BIC
Fixed intercept^1^		10922.59		10926.59	10938.13
Random intercept for^2^					
Survey period	0.01	10913.53	$\chi^{2}$ (1) = 9.06, *p =* 0.003	10919.53	10936.84
Medical school	0.02	10867.23	$\chi^{2}$ (1) = 55.36, *p* < 0.001	10873.24	10890.53
Survey period*medical school	0.04	10862.64	$\chi^{2}$ (1) = 59.95, *p* < 0.001	10868.64	10885.94

*n* = 2,370; ICC, Interclass correlation coefficient, −2 LL = restricted −2 Log Likelihood; LRT, Likelihood Ratio Test; AIC, Akaike Information Criterion; BIC, Bayesian Information Criterion.

1 $Y_{i}=\beta_{0}+\varepsilon_{i}$ .

2 $Y_{\mathrm{ij}}=\beta_{0}+\tau_{i}+\varepsilon_{\mathrm{ij}}$ .

The assumptions of linear mixed modeling were checked and overall met. However, the residuals plotted against the predicted values showed a negative linear trend. This likely reflects the limited, quasi-interval nature of the 9-point scale used for the dependent variable. As a result, the model may systematically overestimate high values and underestimate low values of this variable. Interpretations therefore focus on the direction and significance of effects rather than on their precise magnitude. Professional and academic pre-qualifications (vocational training, voluntary service, academic degree) and interest in artistic-linguistic, entrepreneurial, or orderly-administrative activities did not contribute significantly to the model. However, medical students with a higher interest in practical-technical or social activities and with a lower interest in intellectual-research activities reported a significantly higher interest in working as a rural doctor. Of our control variables older medical students showed more interest in rural practice and having planned or completed an internship in general practice, interest in General Medicine and rural origin predicted interest in rural practice. The control variables gender and semester level showed no significant effect, see Table 5 for the results of the linear mixed model. Estimated marginal means (EMMs) were calculated to illustrate the effects of the predictors. In particular, students with an interest in General Medicine (*M* = 6.44, SE = 0.15) were significantly more interested in working in rural medicine than students without such an interest (*M* = 3.74, SE = 0.15). The differences in the EMMs of interest in working as a rural doctor (scale from 1 to 9 points) between those with and without vocational training (difference = −0.14), between those with and without an academic degree (difference −0.04), and between those with and without voluntary service (difference = 0.08) were very small (see Table 6). As our sample includes medical students who completed several prior qualifications before starting their studies, we calculated the EMMs for all possible combinations of pre-qualifications. Supplementary Figure S2 in the Appendices shows that there was little difference in interest in rural practice between the groups.

**Table 5:** Results of the linear mixed regression analysis (random intercept for survey period*medical school) with interest in working as a rural doctor as dependent variable including coefficients, standard errors, *p*-values, confidence intervals, and fit statistics.

Predictors	B^1^	SE(B)	df	*p*	CI(B)	
					LL	UL
Vocational training (yes)	−0.14	0.12	2186.05	0.255	−0.37	0.10
Academic degree (yes)	−0.04	0.19	2201.98	0.810	−0.41	0.32
Voluntary service (yes)	0.08	0.10	2200.30	0.404	−0.11	0.27
Practical-technical activities	0.07	0.02	2201.18	<0.001***	0.03	0.11
Intellectual-research activities	−0.09	0.02	2202.12	<0.001***	−0.13	−0.05
Artistic-linguistic activities	0.03	0.02	2199.77	0.142	−0.01	0.06
Social activities	0.16	0.03	2197.06	<0.001***	0.11	0.21
Entrepreneurial activities	0.02	0.02	2200.76	0.396	−0.02	0.06
Orderly-administrative activities	0.01	0.02	2200.59	0.492	−0.03	0.05
Gender (male)	−0.17	0.09	2202.52	0.065	−0.35	0.01
Age	0.05	0.02	2199.51	0.003**	0.02	0.08
Semester level^1^						
3rd	0.30	0.19	356.75	0.107	−0.06	0.67
6th	0.27	0.15	2175.46	0.069	−0.02	0.55
10th	0.01	0.12	2188.11	0.964	−0.23	0.24
Internship in general practice^2^						
Planned	0.57	0.12	2202.61	<0.001***	0.34	0.81
Completed	0.50	0.14	2191.66	<0.001***	0.22	0.78
Rural origin (yes)	−0.67	0.09		<0.001***	−0.85	−0.49
Interest in general medicine (yes)	2.70	0.09	2199.28	<0.001***	2.53	2.88
Intercept	3.38	0.19	200.94	<0.001***	3.02	3.75

AIC = 9116.54; BIC = 9127.94, −2LL = 9112.54; var.: level-1 residuals = 3.43; var.: random intercept = 0.04, ICC = 0.01.

CI, confidence interval; LL, lower level; UL, upper level; AIC, Akaike Information Criterion; BIC, Bayesian Information Criterion; −2LL = restricted -2LogLiklihood.

1 The reference category = ‘final year’.

2 The reference category = ‘no general practice internship planned or completed’.

*p < 0.05, ** *p* < 0.01, *** *p* < 0.001.

**Table 6:** Estimated marginal means of interest in rural practice (scale from 1 to 9) for all categorical predictors of the model.

Predictors	*M*	SE	i-j	*n*
Vocational training				
Yes (i)	5.03	0.16	−0.14	617
No (j)	5.16	0.14		1,605
Academic degree				
Yes (i)	5.07	0.20	−0.04	136
No (j)	5.12	0.12		2,086
Voluntary service				
Yes (i)	5.14	0.16	0.08	520
No (j)	5.05	0.14		1702
Gender				
Male (i)	5.01	0.15	−0.17	777
Female (j)	5.18	0.14		1,445
Semester level = 3rd				
Yes (i)	5.24	0.14	0.30	569
No (j)	4.94	0.19		1,653
Semester level = 6th				
Yes (i)	5.22	0.18	0.27	687
No (j)	4.96	0.13		1,535
Semester level = 10th				
Yes (i)	5.10	0.17	0.01	687
No (j)	5.09	0.13		1,757
Rural origin				
No rural origin (i)	4.77	0.14	−0.65	1,620
Yes, rural origin (j)	5.42	0.15		602
Internship in general practice = planned				
Yes (i)	5.38	0.15	0.58	841
No (j)	4.81	0.15		1,381
Internship in general practice = completed				
Yes (i)	5.34	0.17	0.50	977
No (j)	4.84	0.13		1,245
Interest in general medicine				
Yes (i)	6.44	0.15	2.70	763
No (j)	3.74	0.15		1,459

*M* represent estimated marginal means with standard error (SE).

The *post hoc* sensitivity analysis indicated that given the large sample size (*N* = 2,370), *α* = 0.05, power (1-*β*) of 0.90 and 9 tested predictors within a model including 18 covariates, the minimum detectable effect size was *f*^2^ < 0.01, corresponding to a small effect (55). This suggests that even subtle effects, accounting for less than 1% of the total variance in the dependent variable could have been detected. However, as the power analysis was based on a fixed-effects linear regression model, it does not fully account for the multilevel structure of the data. As such, the sensitivity estimation should be interpreted as an approximation. In our analysis, however, statistical significance was not equated with practical relevance. Interpretations focus on theoretically meaningful and consistent patterns.

## 4 Discussion

We conducted a cross-sectional study at five medical faculties in Baden-Wuerttemberg, Germany with an overall sample size of *N* = 2,370 (i.e., 3rd-, 6th-, and 10th semester and final-year students). To get an overview of which specialty training programs are generally of interest to different groups, we investigated the differences between medical students with and without professional pre-qualifications (i.e., vocational training in the medical field, academic degree or voluntary service) (RQ1). We found differences in preferences for different specialties, particularly among medical students who had completed vocational training (vs. not) or voluntary service (vs. not). The strongest association was between vocational training and interest in Anesthesiology, with trained students showing greater interest than those without training. The main focus of our study was to analyze how professional and academic pre-qualifications (RQ2) and different vocational interests according to Holland’s RIASEC model relate to interest in working as a rural doctor (RQ3). We observed no association between having completed vocational training, an academic degree or voluntary service and interest in working as a rural doctor. However, higher interest in practical-technical and social activities and lower interest in intellectual-research activities were associated with a greater interest in rural practice. There were no associations with interest in artistic-linguistic, entrepreneurial or orderly-administrative activities.

### 4.1 Pre-qualifications and differences in specialty training aspirations

There has been little research into how professional and academic pre-qualifications relate to career aspirations (22). Overall, medical students expressed the greatest interest in Internal Medicine, which is consistent with previous findings from Germany (32, 50). In our study, medical students were able to indicate up to three preferences for different specialty training programs. This allows students who are still in the decision-making process to give a more realistic indication of their preferences, but limits the comparability of the frequencies with other studies. Based on this response format, we were able to identify the most frequently reported combination of medical specialties, which was ‘General Medicine–Anesthesiology–Internal Medicine’.

To answer RQ1, the three pre-qualifications we defined were considered separately. In line with previous studies, medical students who had completed vocational training in the medical field were more likely to be interested in General Medicine (6, 32) or Anesthesiology (32). As a large proportion in our sample of students with completed vocational completed paramedic training, this may explain the high interest in Anesthesiology, as both paramedics and anesthesiologists are exposed to emergency situations (56–58). This may be particularly relevant for Germany, where professional experience and vocational training in the medical field are highly valued in the admissions system compared to other countries (21). As a result, a relatively large number of people in Germany train as nurses or paramedics before studying medicine. In addition, the ‘waiting list quota’ was in place until 2020, and many students bridged the waiting period for a place at medical school with vocational training (59).

Our results show similar preferences for students with vocational training and those who have completed voluntary service (higher interest in Anesthesiology, General Medicine and lower interest in Ophthalmology, Otorhinolaryngology, Surgery). The higher interest in General Practice that we found among this subgroup is consistent with studies linking volunteering or social/societal orientation to increased interest in general (rural) practice (17, 18, 46, 60). For medical students with a prior academic degree, only two significant associations emerged. They showed a higher interest in ‘other’ specialties and lower interest in Pediatrics than those without a prior degree. One possible explanation is that previous study experience may not have been specific enough to influence interest in different specialties, so that medical students with academic degree may differ only slightly from those without.

Our rationale for this study was that medical students who have completed professional or academic qualifications prior to medical school may have different career preferences to their peers who have not. We were able to identify such differences for some specialty training programs and depending on the type of pre-qualification. Based on Social Learning Theory (26) and theories of the development of specialty preferences (30, 31), it was hypothesized that prior learning experiences would influence identity and career formation. The Community of Practice (CoP) Model (61, 62) offers another possible theory of how experiences shape identity and thus influence life and career choices. The model emphasizes the social component of learning and describes a CoP as a group of people who share a common interest and knowledge and exchange ideas about it (61, 62). The three characteristics of a CoP are (1) the domain of interest which creates a common identity in the group, (2) the community for which the domain is relevant and (3) the practice as the knowledge and methods shared (61). Medical students who have completed vocational training in the medical field prior to medical school, or who have had other academic or practical experience, may have participated in a CoP (or in its periphery) even before entering medical school. Through practical experience and interactive learning processes, a CoP with a shared identity and a common understanding of the values and ways of working in its environment may have developed. The model may explain the differences we found. When students start their medical studies, they gradually become part of a (new) CoP, which is based on or influenced by previously existing CoPs.

### 4.2 Pre-qualifications, vocational interests and rural doctor aspirations

In our study, we found no association between professional and academic pre-qualifications and interest in working as a rural doctor (RQ2). Neither having completed a vocational training in the medical field, an academic degree nor a voluntary service could be associated with interest in rural practice. Our results contradict those of Kesternich et al. (35), who found an association between pre-study work experience and interest in rural practice However, the authors only found small effects at a significance level of 10%.

We found no studies on the relationship between a previous academic degree and interest in rural practice, so our findings make an important contribution here. The fact that we found no differences between medical students who did and did not complete voluntary service contradicts the findings of Feldmann et al. (17), who found an association between having completed a voluntary service and a greater interest in rural medicine. While both interest in rural medicine and prior voluntary service/vocational training could be related to interest in General Medicine in prior studies (17, 35), we found no direct relationship between interest in rural medicine and professional and academic pre-qualifications. As part of our first research question (RQ1), we were able to show that the specialty preferences of people with previous experience may differ from those of people without previous experience. However, this does not seem to be the case for interest in working as a rural doctor. The professional and academic pre-qualifications we have defined may be too imprecise to influence interest in rural practice, and further studies could explicitly analyze previous experiences in rural areas.

As part of our analysis, we also looked at different control variables. While previous studies have shown mixed results regarding the influence of gender (16, 63), we found no differences between men and women regarding interest in rural medicine. We also found no differences between the different semester levels in our sample. However, consistent with previous studies, our control variables (higher) age (16, 17), rural origin (6, 16), interest or contact with General Medicine (33) were positively associated with interest in rural practice. The relationship with interest in General Medicine was by far the strongest in the overall model and recommendations for medical education can be derived from this. Medical schools could strengthen their profile in General Medicine. In addition, specific courses and placements in rural areas could be offered to encourage interest in general practice and working in rural areas. Again, the COP model can provide important impetus for practical recommendations. Medical schools could specifically promote the development of CoPs in general and rural medicine as part of their curricula (64). Practical experience (e.g., through internships) plays a particularly important role here, as learning is not only about acquiring knowledge, but also about increased participation in CoPs (61, 65).

Regarding RQ3, higher interest in practical-technical activities, higher interest in social activities and lower interest in intellectual-research activities were associated with higher interest in working as a rural doctor. There were no associations with an interest in artistic-linguistic, entrepreneurial or orderly-administrative activities. Previous studies showed a link between the social (41, 42, 45) and investigative (41, 45) components of Holland’s RIASEC model and interest in general practice. Based on these findings we expected similarities in the RIASEC profile for rural practice. Our findings on the social (S) Holland component are consistent with studies showing an association between social orientation and rural practice (17, 18). However, in our sample, interest in intellectual-research activities was negatively associated with interest in rural practice. This is consistent with a study of motivational factors influencing interest in rural medicine, which showed that students motivated by intellectual challenge were less interested in rural practice (33). Instead, we found a positive relationship with interest in practical-technical activities. A study on the RIASEC types and medical specialties shows that Holland’s type R (realistic = practical-technical) can be mapped to the surgical discipline (43). The authors used the R component to describe surgeons as a group with high practical skills, craftsmanship and efficient outcomes. This may also be relevant for rural doctors. Far from larger cities with specialists for specific conditions, rural doctors may have to deal with a wide range of tasks and medical conditions that may require a more practical approach. Therefore, medical students who are more interested in practical-technical activities may be more interested in rural work, or vice versa. In order to stimulate interest in rural medicine, interest in social and practical activities could be encouraged within the medical curriculum or even made a priority for admission to medical school. Interestingly, our sample of medical students showed a high level of interest in social activities compared to the other RIASEC dimensions, which is in line with other findings on vocational interests within medical students (42). Given the shortage of rural doctors, selection procedures could place more emphasis on social and communication skills (e.g., emotional availability) in the medical school admission process (66).

The findings of this study contribute to a more comprehensive understanding of the factors associated with interest in working as a rural doctor. However, most of the effects we found were small indicating important trends, but suggesting that there may be other influencing factors that were not investigated in our study. Career development and the choice of specialty program are complex and may be influenced by many factors, including personality traits (67), gender differences (68, 69), work and time-related aspects, career-related aspects and patient orientation (69), and also the specific home-country (68). Career paths taken by medical students, may also be significantly influenced by unplanned events, chance and the nonlinearity of change (70, 71).

Holland’s RIASEC model (36) offers a way to match medical students’ specialty preferences with their vocational interests. However, the model has its limitations. While individuals tend to seek a match between their interests and their environment (36), an optimal match is not always necessary. It is important that characteristics that are personally important to the individual are present in the work environment (72). Further research into the factors that may prevent medical students from considering working in rural areas, and how existing barriers can be overcome (3, 5).

### 4.3 Strengths and limitations

In the present large study, conducted at five medical schools in Germany, we examined professional and academic pre-qualifications and vocational interests in relation to the career aspirations of medical students. To our knowledge, no other study has examined these relationships in such a broad way. Our findings contribute to a better understanding of potential factors influencing interest in rural medical practice and to the crucial issue of securing primary care, especially in rural Germany (73).

However, there are some limitations to mention. First, there is no consistent definition of ‘rural’ in the literature. Rural practice in Central Europe has a different meaning than in large scale countries such as Australia or the USA. Second, our cross-sectional study only captures interests and not actual career choices, so further longitudinal studies are needed to identify factors that influence the process of specialty choice. Furthermore, when interpreting the results of the dichotomous variables ‘vocational training’, ‘academic degree’ and ‘voluntary service’, the following should also be taken into account: Firstly, the three variables do not include students who dropped out before completing the respective qualification. Secondly, each dummy variable should be interpreted independently, as it is also possible that a person with vocational training has previously done voluntary work before. Also due to the quasi-interval nature of our dependent variable ‘interest in rural practice’, interpretations of the linear mixed model results should focus on the direction and significance of the effects rather than on their precise magnitude. Finally, our study was conducted across all medical faculties in the German state of Baden-Württemberg and is not representative of medical schools in Germany in general, and as participation in the study was voluntary, potential selection bias cannot be ruled out.

## 5 Conclusion

Due to the German admissions system for medical studies, there is a relatively large group of medical students who have already gained practical experience before medical school (21, 22). However, there has been little research on this group of ‘pre-experienced’ medical students in general and their career aspirations in particular (22). Our study showed that specialty preferences may differ according to prior experience. This finding is relevant to researchers in the field of medical education, as it means that prior experience may lead to the formation of a professional identity and community of practice (CoP) even before starting medical school. However, we were also able to show that professional and academic pre-qualifications did not seem to have any influence on interest in working as a rural doctor. We were able to identify other influencing factors, such as rural origin, interest in or placements in general practice and interest in social or practical-technical activities. The results of this study suggest that medical schools should promote interest in rural medicine through targeted curricular adjustments, such as rural placements, and place more emphasis on general practice and encourage the development of rural practice CoPs at an early stage. Emphasizing interprofessional training and collaboration as part of a team approach to patient care is also important and should be encouraged in modern healthcare systems (74). In addition to the factors influencing interest in rural practice, we recommend longitudinal research into concepts such as the rural doctor quota introduced in Germany (6), the training of medical assistants (75) and telemedicine (75, 76).

## Data Availability

The datasets presented in this article are not readily available because of the high confidentiality of the data. The authors of the study received permission from the Medical Faculty of Tübingen to conduct the study and to collect these data only if they were not made publicly available without individual permission for specific questions (i.e. on request). Requests to access the datasets should be directed to psychosomatik@med.uni-tuebingen.de.
