# Supplementary material for: Professional and academic pre-qualifications, career preferences and aspirations in working as a rural doctor
Source: Front Med (Lausanne). 2025 Jul 9;12:1566303. doi: 10.3389/fmed.2025.1566303 (PMC12284000; doi:10.3389/fmed.2025.1566303)
Supplement: Supplementary file 1 [file Image_1.pdf]

Figure S1

Specialty preferences separately for each semester level in percentages

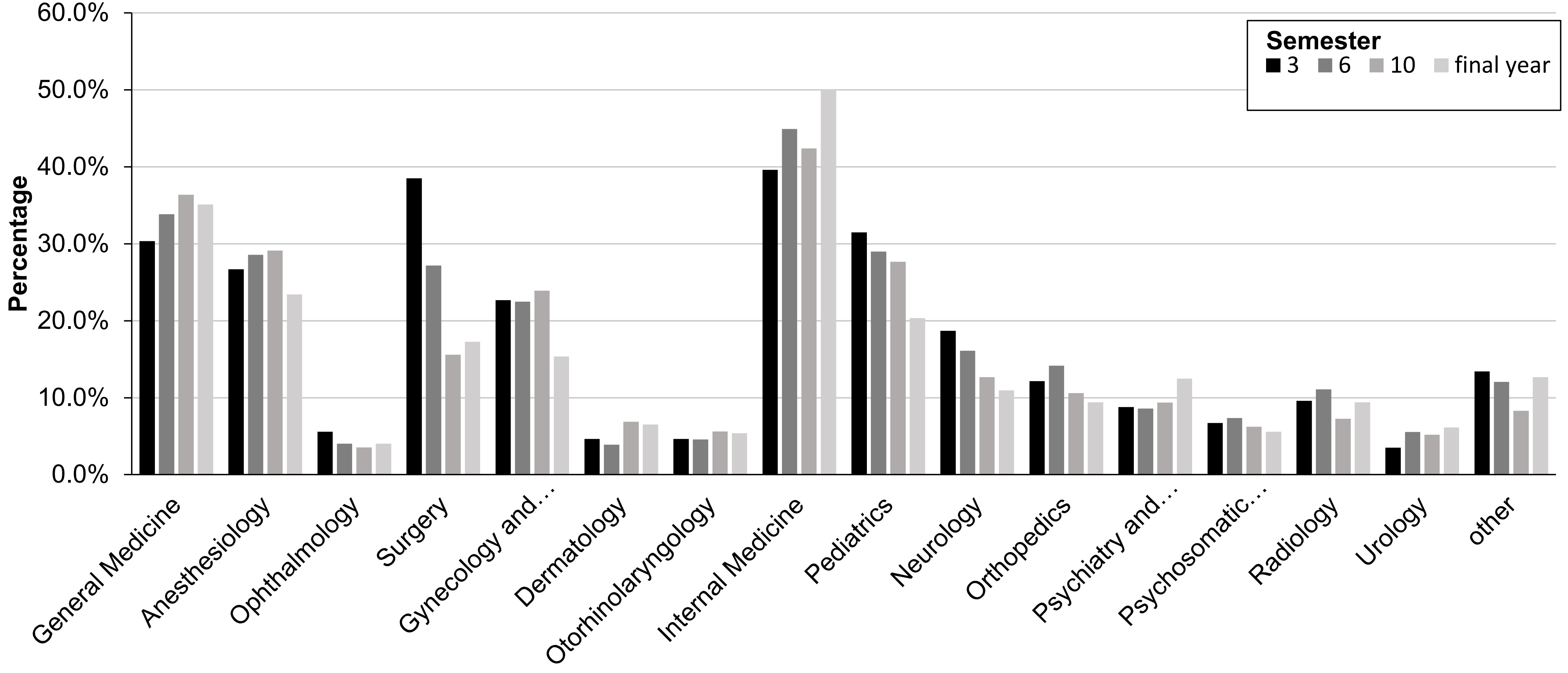

Note. For each semester, the percentage of students who expressed an interest in the 15 specialties (+ 'other' specialty) was calculated separately. Interpretation example: 30.4% of all students in the third semester expressed an interest in General Medicine (first bar, black). Medical students could list up to three preferences
