# Supplementary material for: Professional and academic pre-qualifications, career preferences and aspirations in working as a rural doctor
Source: Front Med (Lausanne). 2025 Jul 9;12:1566303. doi: 10.3389/fmed.2025.1566303 (PMC12284000; doi:10.3389/fmed.2025.1566303)
Supplement: Supplementary file 2 [file Image_2.pdf]

**Figure S2**

*Estimated marginal means (EMMs) of interest in rural practice for all possible combinations of professional and academic pre-qualifications (i.e. vocational training, academic degree, voluntary service)*

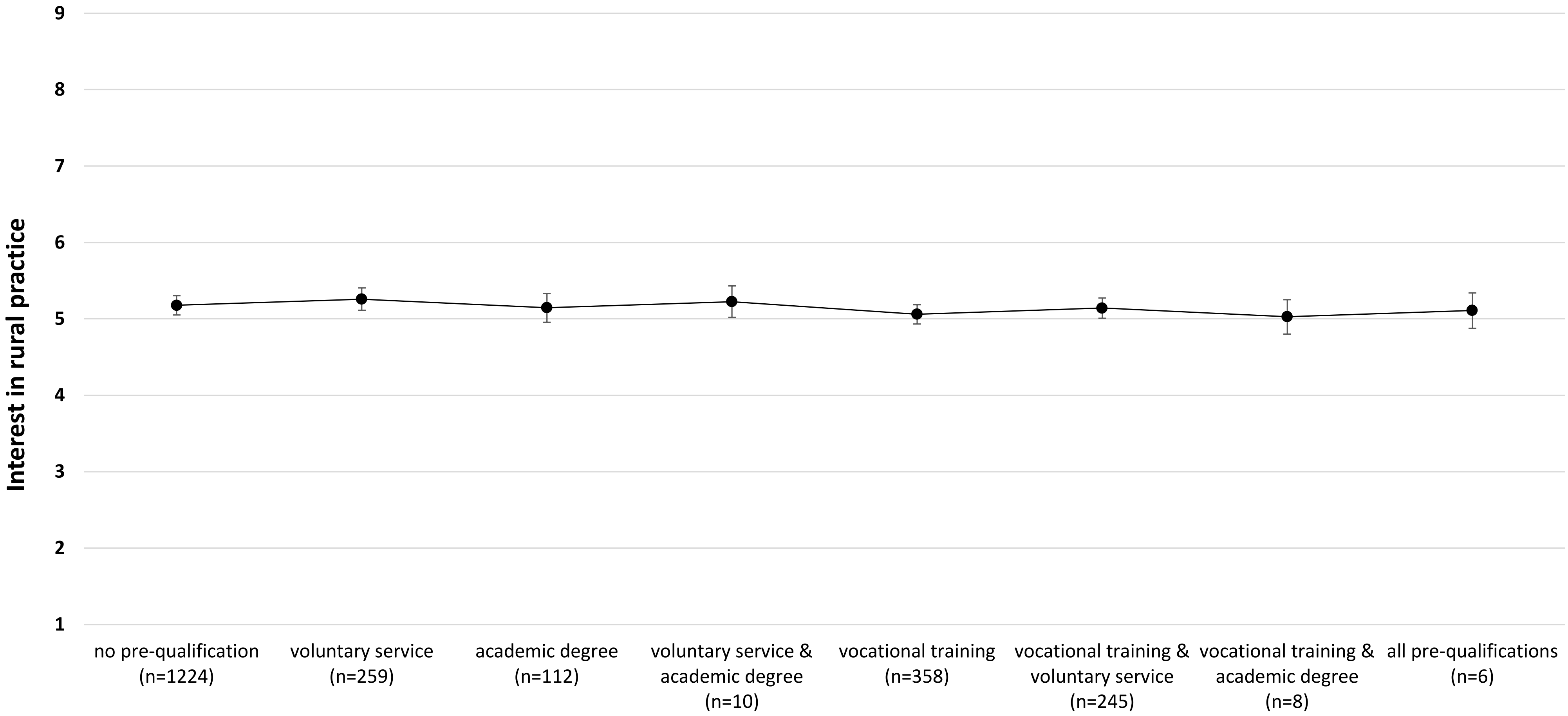


*Note.* Error bars represent standard errors of the EMMs
