# Supplementary material for: Professional and academic pre-qualifications, career preferences and aspirations in working as a rural doctor
Source: Front Med (Lausanne). 2025 Jul 9;12:1566303. doi: 10.3389/fmed.2025.1566303 (PMC12284000; doi:10.3389/fmed.2025.1566303)
Supplement: Supplementary file 3 [file Table_1.docx]

**Table S1**

*List of the most frequently (≥1.0%) indicated combinations of specialist training programs*

| no. | combination |  | *n* |  | % |
| --- | --- | --- | --- | --- | --- |
|  |  |  |  |  |  |
| 1 | General Medicine;Anesthesiology;Internal Medicine |  | 84 |  | 3.6 |
| 2 | General Medicine;Internal Medicine;Pediatrics |  | 54 |  | 2.3 |
| 3 | Anesthesiology;Internal Medicine |  | 46 |  | 2.0 |
| 4 | Anesthesiology;Internal Medicine;Pediatrics |  | 42 |  | 1.8 |
| 5 | General Medicine;Gynecology and Obstetrics;Internal Medicine |  | 38 |  | 1.6 |
| 6 | General Medicine;Gynecology and Obstetrics;Pediatrics |  | 38 |  | 1.6 |
| 7 | Pediatrics |  | 38 |  | 1.6 |
| 8 | Internal Medicine |  | 36 |  | 1.5 |
| 9 | General Medicine;Internal Medicine;Neurology |  | 33 |  | 1.4 |
| 10 | Anesthesiology;Surgery;Internal Medicine |  | 32 |  | 1.4 |
| 11 | Gynecology and Obstetrics;Internal Medicine;Pediatrics |  | 27 |  | 1.1 |
| 12 | Surgery |  | 27 |  | 1.1 |
| 13 | General Medicine;Internal Medicine |  | 26 |  | 1.1 |
| 14 | Anesthesiology;Surgery;Orthopedics |  | 25 |  | 1.1 |
| 15 | Surgery;Internal Medicine;Neurology |  | 25 |  | 1.1 |
| 16 | other |  | 24 |  | 1.0 |
| 17 | Surgery;Internal Medicine;Pediatrics |  | 24 |  | 1.0 |
| 18 | General Medicine;Anesthesiology;Pediatrics |  | 23 |  | 1.0 |
| 19 | Internal Medicine;Pediatrics |  | 23 |  | 1.0 |
|  | … |  | … |  | …. |
| 445 | Overall |  | 2349 |  | 100.0 |

*Note.* Medical students were able to list up to three preferences.
